# The legend of modern hysteroscopic surgery ^*^: ^*^ Video Article, to see the video use this link: https://qrco.de/bbSLLG

**Published:** 2020-08-05

**Authors:** Attilio Di Spiezio Sardo

Hysteroscopy is generally considered the Copernican revolution of modern gynaecology. Nicolaus Copernicus, a Polish astronomer, was the one who changed the Tolemaic model of the universe, which had the earth stationary at the centre of the universe, to the Heliocentric model, with the sun at the centre surrounded by all the other planets. It’s not an exaggeration to say that Copernicus and this revolution fundamentally changed the way we think about the place of human beings in the universe.

In a similar way, hysteroscopy changed the way of approaching the uterine cavity. Hysteroscopy, differently to laparoscopy, which simply modified the access to the abdominal cavity and to the outer surface of uterus, has for the first time put the sun, the light into a cramped and dark space, which was never directly explored until the mid-19^th^ century.

However, in its early days, hysteroscopy was considered the “Cinderella” subspecialty of gynaecological endoscopy, due to the challenge of obtaining a satisfactory distention of the uterine cavity to allow adequate visualization. This was the reason why hysteroscopy was rarely used in routine clinical practice, which was mostly based on blind “dilatation and curettage” for both the diagnosis and treatment of intrauterine pathologies.

Although some scientific societies and authors have persistently continued to emphasize the diagnostic and therapeutic role of D&C, a large number of papers have extensively shown throughout the years the significant limitations of this technique: 1) the need for in-patient admission and general or regional anaesthesia; 2) the high risk of complications (such as perforation, intrauterine adhesion formation, infections); 3) poor diagnostic accuracy (related to the high number of focal lesions easily missed); and the total absence of any therapeutic role (Word et al., 1958; Bettocchi et al., 2001).

Over the past 40 years, the higher diagnostic accuracy due to its ability of direct endoscopic visualization together with many important and significant technical and technological innovations have opened new horizons for hysteroscopy, which is today considered the gold standard for the diagnosis and treatment of intrauterine pathologies.

Some dates are important in the modern history of hysteroscopy; The year 1959 is one of them. It was an important year as 1) the cold war was ending and the meeting between the Soviet Leader Khruschev and the President of the United States Eisenhower was very important; 2) the Prince of Japan married a commoner girl, expressing the post-war democratization of the palace ; 3) the loss of independence of Tibet by Chinese Army which forced the Dalai Lama into exile; 4) the triumphant entry of Fidel Castro in Havana at the helm of his loyal “barbudos” on January 1^st^.

The year 1959 also represents the birth of Hopkins’ rod lens optic in hysteroscopy, thanks to the collaboration between Professor Harold Horace Hopkins with the German inventor Karl Storz. They modified the shape and length of the lens inside the optics: from a small and spherical lens to longer and cylindrical one. This resulted in an inverted ratio of air to lens in favour of the lenses which provide optics with higher image definition.

In the 1980s a revolution occurred in hysteroscopy, thanks to the great man and doctor Jacques Hamou and his extraordinary inventions in every field of hysteroscopy (Hamou, 1990). There are some important points in Dr. Hamou’s biography that deserve to be pointed out. He first studied mathematics and physics before deciding to study medicine. Once starting his medical studies, he became fascinated with hysteroscopy and went to the United States to learn the technique. There he was filled with a desire to make this procedure ambulatory. So he came back to Paris and, using his knowledge of physics, he started to work to develop a new instrument with the German inventor Karl Storz; a new hysteroscope that would revolutionize the world of hysteroscopy at that time. The Hamou I microcolpohysteroscope had a diameter of 5 mm and allowed four different powers of magnification up to 150 times magnification for cellular exploration.

Hamou also described carbon dioxide distension and the fore-oblique view of the optic, which enabled the visualisation of the entire uterine cavity only by rotating the scope without painful lateral movements. These helped the transformation of hysteroscopy into a truly ambulatory procedure. Then, he published a book on hysteroscopy and microcolpohysteroscopy which became a guide for those who were attracted to the technique all over the world (Hamou, 1990).

The 90s were the years in which a young Italian doctor, Stefano Bettocchi completely changed the way of approaching intrauterine pathologies. The main technical and technological innovations linked to his name are: 1) the development of oval profile small diameter hysteroscopes with continuous flow features and operative sheaths, through which miniaturized instruments could be inserted; 2) the introduction of the vaginoscopic approach (Bettocchi et al., 1997); 3) the use of miniaturized instruments (mechanical and electrical) to treat pathologies in the office setting (the so called see & treat approach) (Bettocchi et al., 2002) and last but not least the development of an innovative system for fluid distention of the uterine cavity.

Other surgeons who also contributed to the birth of modern hysteroscopy are:

- Ivan Mazzon from Rome, Italy, who developed the cold loop myomectomy technique for the treatment of challenging submucosal myoma with intramural component (Mazzon et al., 2014);
- Mark Hans Emanuel from the Netherlands, who was the first to develop a tissue removal device for the mechanical removal of intrauterine pathologies in a faster and easier way without the use of electrosurgery (Emanuel et al.,2005);
- Giampietro Gubbini from Bologna, Italy who developed the miniresectoscope which adopted the technical rules and surgical manoeuvres of traditional resectoscopic surgery and applied them to ambulatory surgery (Dealberti et al., 2016);
- Rudi Campo from Belgium who had a great influence on the miniaturisation of hysteroscopes with his revolutionary Trophyscope with an outer diameter of only 2.9 mm for diagnostic procedures that can be turned into a 4.4 mm operative hysteroscope by simply sliding forward an accessory sheath (El-Toukhy et al., 2016). The availability of the Trophyscope together with other versatile operative instruments in an outpatient clinic where 3D sonography is integrated into the endoscopic tower represents the birth of a new concept “The Hysteroscopy Digital Clinic” (Campo et al., 2018).

And last but not least, Luis Alonso Pacheco and Sergio Haimovich, who with their passion and determination have contributed to an incredible world-wide diffusion of hysteroscopy in recent years and its establishment as an independent gynaecological procedure separate from laparoscopy.

However, the complexity of modern hysteroscopy has also increased the demand and brought new challenges to surgical education and quality control. Clinicians who perform endoscopic surgery without proper training are at a higher risk of having complications, increasing the patient morbidity and mortality. Professional organizations are responsible for setting the standards for training the next generation of endoscopists to ensure patient safety.

In this light, the GESEA programme, developed by the European Society for Gynaecological Endoscopy, represents a unique opportunity for training and certification in endoscopic surgery.

In conclusion, the history of hysteroscopy was written by many individuals and their incredible minds that have made it possible to conceive and develop countless increasingly advanced tools and techniques so that, with proper training, we are now able to treat most of the intrauterine pathologies with safety and efficacy. There is no doubt that the progress will not end here, we are bound to see further episodes of this legend.

## Video scan (read QR)

https://qrco.de/bbSLLG

**Figure:**
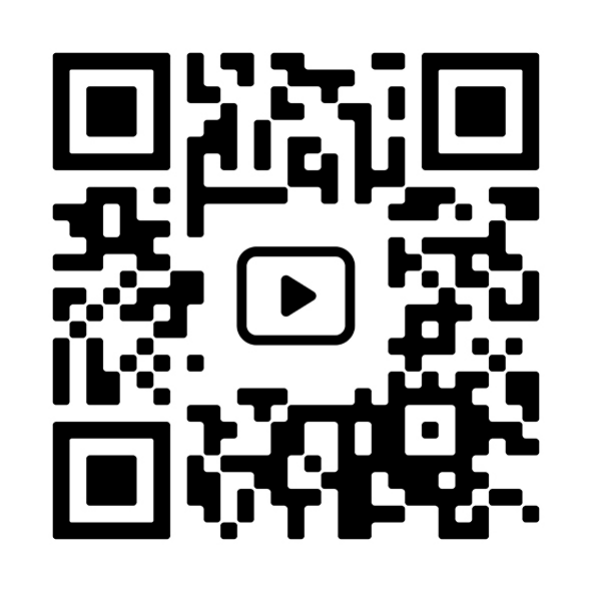


Attilio Di Spiezio Sardo, Full Professor of Obstetrics & Gynecology, Department of Public Health, University Federico II of Naples, Italy
